# Pressure effect on iron-based superconductor LaFeAsO_1−*x*_H_*x*_: Peculiar response of 1111-type structure

**DOI:** 10.1038/srep39646

**Published:** 2016-12-22

**Authors:** Kensuke Kobayashi, Jun-ichi Yamaura, Soshi Iimura, Sachiko Maki, Hajime Sagayama, Reiji Kumai, Youichi Murakami, Hiroki Takahashi, Satoru Matsuishi, Hideo Hosono

**Affiliations:** 1 Institute of Materials Structure Science, High Energy Accelerator Research Organization (KEK), Tsukuba, Ibaraki 305-0801, Japan; 2 Materials Research Centre for Element Strategy, Tokyo Institute of Technology, Yokohama, Kanagawa 226-8503, Japan; 3 Laboratory for Materials and Structures, Tokyo Institute of Technology, Yokohama, Kanagawa 226-8503, Japan; 4 Department of Materials Structure Science, The Graduate University for Advanced Studies, Tsukuba, Ibaraki 305-0801, Japan; 5 College of Humanities and Science, Nihon University, Setagaya, Tokyo 156-8550, Japan

## Abstract

A systematic study of the crystal structure of a layered iron oxypnictide LaFeAsO_1−*x*_H_*x*_ as a function of pressure was performed using synchrotron X-ray diffraction. This compound exhibits a unique phase diagram of two superconducting phases and two parent phases. We established that the As–Fe–As angle of the FeAs_4_ tetrahedron widens on the application of pressure due to the interspace between the layers being nearly infilled by the large La and As atoms. Such rarely observed behaviour in iron pnictides implies that the FeAs_4_ coordination deviates from the regular tetrahedron in the present systems. This breaks a widely accepted structural guide that the superconductivity favours the regular tetrahedron, albeit the superconducting transition temperature (*T*_c_) increases from 18 K at ambient pressure to 52 K at 6 GPa for *x* = 0.2. In the phase diagram, the second parent phase at *x* ~ 0.5 is suppressed by pressure as low as ~1.5 GPa in contrast to the first parent phase at *x* ~ 0, which is robust against pressure. We suggest that certain spin-fluctuation from the second parent phase is strongly related to high-*T*_c_ under pressure.

Iron pnictides are a new family of high-temperature superconductors, whose charge carriers located primarily in the two-dimensional iron plane play an active role in its superconductivity^1^,^2^,^3^,^4^. The supporting pnictide (*Pn*) atoms strongly perturb the 3*d* multi-orbital bands of Fe atoms through hybridization between Fe-3*d* and *Pn*-*p* electrons^2^,^3^,^4^. As a consequence, chemical substitution along with carrier-doping/chemical-pressure, or application of pressure on the crystal can drastically modify electronic properties, such as the superconducting transition temperature (*T*_c_) of these materials^2^,^3^,^4^,^5^. Based on studies on chemical substitution in iron pnictides, an empirical guideline has been established that *T*_c_ is maximised when the geometry of the Fe*Pn*_4_ unit approaches a regular tetrahedron^6^. The application of pressure is a direct and clean way to modify the local geometry of Fe*Pn*_4_ without the degradation of the crystal in comparison to the chemical substitution; hence, the detailed crystal structure under pressure warrants further investigation.

One of the fascinating materials in iron pnictides is LaFeAsO_1−*x*_H_*x*_, which has a ZrCuSiAs-type structure with alternating stacks of conducting FeAs_4_ moieties and insulating (O, H)La_4_ layers (Fig. 1a) 7,8. LaFeAsO_1−*x*_H_*x*_ exhibits a unique phase diagram on hydrogen anion substitution *i.e.* electron doping: two superconducting domes with *T*_c,max_ = 26 K at *x* ~ 0.08 (SC1) and *T*_c,max_ = 37 K at *x* ~ 0.35 (SC2), and two parent phases at *x* ~ 0 (PP1) and *x* ~ 0.5 (PP2)^9^,^10^,^11^,^12^. The SC2 and PP2 are rarely observed among high-*T*_c_ materials because they usually become normal metal in the heavily electron-doped region^11^. The resistivity behaviour changes from Fermi-liquid to non-Fermi liquid types with doping the electron, implying the electronic correlation developed in the higher *x* region^7^. Takahashi *et al*. have recently demonstrated that the application of pressure on LaFeAsO_0.72_H_0.18_ induced a notable enhancement of the *T*_c_ from 18 K at ambient pressure to 52 K at 6 GPa^13^. Interestingly, the maximum *T*_c_ of LaFeAsO_0.72_H_0.18_ under pressure was similar to that of Sm1111 (55 K), the highest known *T*_c_ among iron-based superconductors^14^.

Here, we examine the crystal structure of LaFeAsO_1−*x*_H_*x*_, (*x* = 0.0, 0.2, and 0.51), under pressure to analyse the relation between the FeAs_4_ geometry and the *T*_c_. The results reveal that the FeAs_4_ unit deviates from regular tetrahedron on the application of pressure. This is an unexpected finding that breaks the hitherto believed guideline of approaching a regular Fe*Pn*_4_ tetrahedron with an increase in *T*_c_. Furthermore, we demonstrate that the peculiar PP2 is rapidly suppressed by pressure, while the conventional PP1 is robust against pressure^15^,^16^,^17^. We suggest that strong spin-fluctuations, originating from an orbital-selective Mott state in the PP2, is a key for enhancing the *T*_c_ under pressure. The pressure responses of the FeAs_4_ modification, the parent phases, and their correlation are previously unexplained peculiarities in 1111-type iron pnictides.

## Results and Discussion

**Figure 1:**
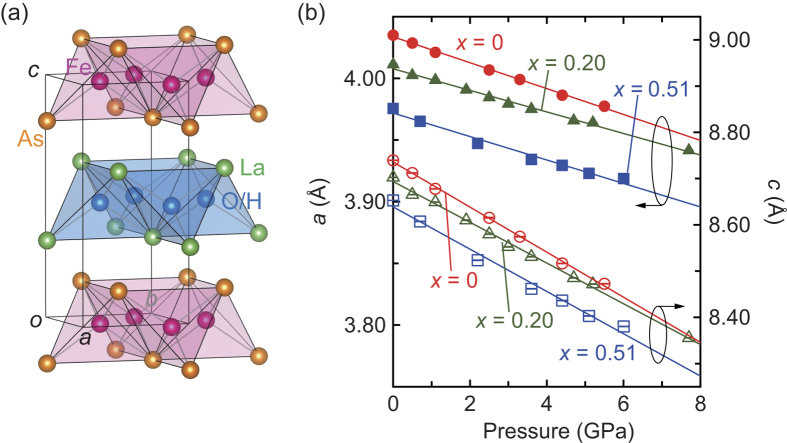
Crystal structure and lattice constants of LaFeAsO_1−*x*_H_*x*_. (**a**) Crystal structure of LaFeAsO_1−*x*_H_*x*_. (**b**) Lattice constants as a function of pressure for *x* = 0, 0.2, and 0.51. The error bars represent the uncertainty in the least-squares fitting of the whole patterns in (**b**).

Figure 1b illustrates the lattice constants (*a* and *c*) of LaFeAsO_1−*x*_H_*x*_ for *x* = 0, 0.20, and 0.51 as a function of pressure at room temperature (detailed experimental data in Supplementary Information, Table S1). No peak broadening was observed over the entire pressure range, indicating that the tetragonal systems are preserved (Supplementary information, Fig. S1a). For each value of *x*, the lattice parameters *a* and *c* decrease monotonically up to ~8 GPa with linear compressibilities of *k*_*a*_ = 2.54–2.62 × 10^−3^ GPa^−1^ and *k*_*c*_ = 5.50–5.64 × 10^−3^ GPa^−1^. These values agree with previously reported linear compressibilities of LaFeAsO_0.72_H_0.18_ and SmFeAsO^13^,^18^. *k*_*a*_ and *k*_*c*_ for all LaFeAsO_1−*x*_H_*x*_ complexes are less than and similar to the respective corresponding values of 3.5 × 10^−3^ GPa^−1^ and 5.4 × 10^−3^ GPa^−1^ for BaFe_2_As_2_^19^. The bulk moduli (*B*_0_) at all values of *x* are estimated as 100(1) GPa using the empirical Murnaghan equation of state (EOS): *V*/*V*_0_ = (1 + *p(B*’_0_/*B*_0_))^−1/*B*’0^, where *V*_0_ is the volume at ambient pressure and *B*_0_’ is fixed at 4.2^20^.

**Figure 2:**
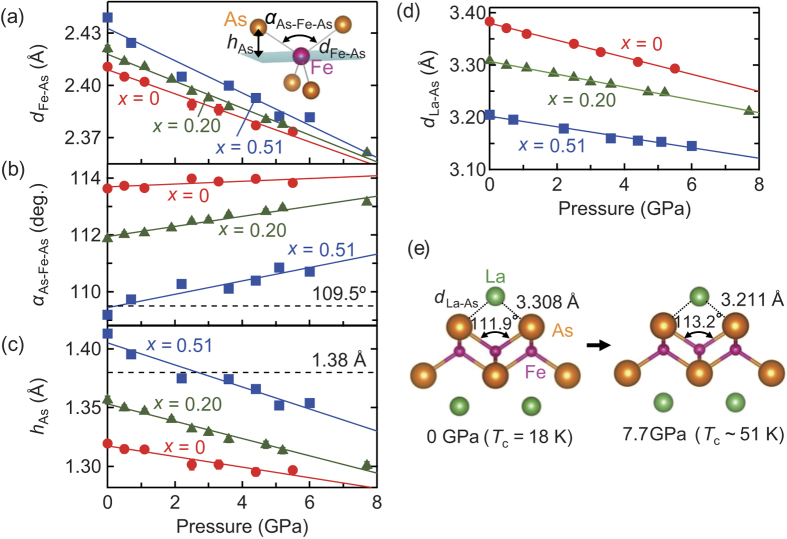
Pressure dependence of structural parameters for LaFeAsO_1−*x*_H_*x*_. (**a**) Fe–As bond length (*d*_Fe-As_). (**b**) As–Fe–As bond angle (*α*_As-Fe-As_). (**c**) As height (*h*_As_). (**d**) La–As distance (*d*_La-As_). The inset in (**a**) describes the FeAs_4_ tetrahedron with the geometrical parameters. The marks are represented for *x* = 0 (red circles), 0.20 (green triangles), and 0.51 (blue squares). Almost all error bars are less than the size of the marks. (**e**) Modification of FeAs_4_ from ambient pressure (0 GPa) to 7.7 GPa for *x* = 0.20. The error bars represent the uncertainty in the least-squares fitting of the whole patterns in (**a**–**d**).

The atomic positions are displayed in Fig. S1b (Supplementary Information). In Fig. 2a–c the Fe–As bond length (*d*_Fe−As_), As–Fe–As bond angle (*α*_As-Fe-As_), and As height from the Fe plane (*h*_As_) are plotted as a function of pressure, where the parameters are described in the inset of Fig. 2a (detailed experimental data in Supplementary Information, Table S1). On the application of pressure, *d*_Fe-As_ and *h*_As_ decrease, while *α*_As-Fe-As_ increases *i.e.* opens up for all the compositions. The degree of pressure response of *α*_As-Fe-As_ is calculated to be +0.049, +0.18, and +0.24°/GPa for *x* = 0, 0.20, and 0.51, respectively. In contrast, that of *α*_As-Fe-As_ in BaFe_2_As_2_ and LiFe_2_As_2_ has been estimated at −0.48 and −0.29°/GPa, respectively, implying that their *α*_As-Fe-As_ bond-angles reduction with pressure^21^,^22^.

Pressure works primarily to infill the interspace between the FeAs_4_ conduction layer and the (O, H)La_4_ insulating layer. Figure 2d displays the La–As distances (*d*_La-As_). Based on previous reports^23^,^24^, the sum of the radii of La and As ions was found to be 3.25 Å, which provides an estimate of the interspace distance. *d*_La-As_ and *α*_As-Fe-As_ change from 3.308(2) Å and 111.9(1)° at ambient pressure to 3.211(1) Å and 113.2(1)° at 7.7 GPa for *x* = 0.20, respectively (Fig. 2e). As *d*_La-As_ for *x* = 0.20 is close to the interspace filling limit, the pressure response of *α*_As-Fe-As_ results in a greater shift at higher *x*. Though the hard sphere perspective is simple, it can yield important findings with regard to superconductivity, but it has not been defined in explicit detail previously in the La1111 system^17^,^25^. Furthermore, as *h*_As_ can be calculated as: *h*_As_ = *d*_Fe-As_cos(*α*_As-Fe-As_/2), it also responds to pressure and decreases significantly in the higher *x* region. On the other hand, in 122-type iron arsenides with a ThCr_2_Si_2_ structure, the drastic reduction of the As–As distance between the FeAs_4_ layers generally causes a structural collapse with the formation of an (As–As)^4−^ molecule^19^,^21^, which possibly drives the reduction of *α*_As-Fe-As_. This distinguishable response of 1111 and 122 compounds to pressure is attributed to the absence of an intervening blocking layer between two FeAs_4_ layers in the latter class of iron arsenides.

**Figure 3:**
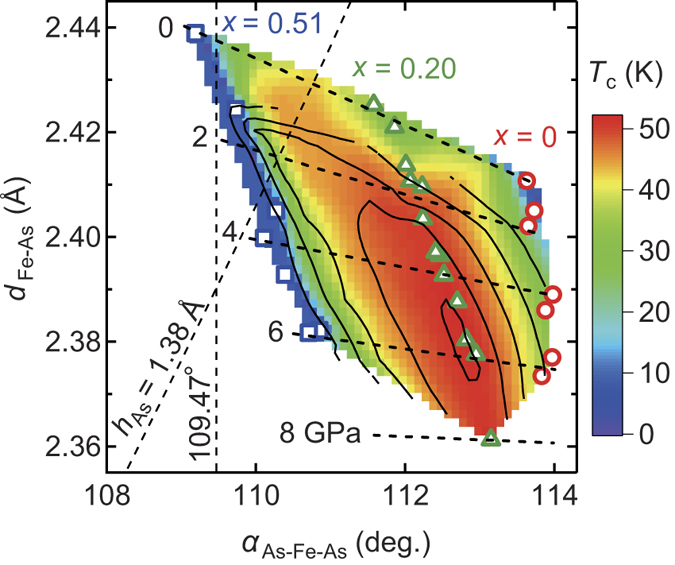
Contour plots of *T*_c_ for LaFeAsO_1−*x*_H_*x*_ as a function of the As–Fe–As bond angle (*α*_As-Fe-As_) and the Fe–As distance (*d*_Fe-As_). Broken lines represent the regular tetrahedron and the As height (*h*_As_) = 1.38 Å in FeAs_4_.

Figure 3 exhibits the contour plots of *T*_c_ against *α*_As-Fe-As_ and *d*_Fe-As_ under pressure, where the values of *T*_c_ in the whole map are interpolated from ref. 13. Since the thermal effect is small when compared to the pressure, it is valid to discuss the relation between the *T*_c_ and the structural parameters at room temperature. Pressure triggers the merge of the two SC domes at ambient pressure into a single SC dome along with an increase of *T*_c_ to 52 K at 6 GPa for *x* = 0.2. After the merge, the ridge line of *T*_c_ runs along the line for *x* = 0.20 as the pressure is increased.

In iron-based superconductors, the relation between the maximum *T*_c_ and structural parameters of FeAs_4_ has been proposed as follows: the highest *T*_c_ is achieved when *α*_As-Fe-As_ approaches 109.5° as in a regular tetrahedron of FeAs_4_ or when *h*_As_ ~ 1.38 Å^6^,^26^. Theoretical argument has been advanced that antiferromagnetic spin- or orbital-fluctuation is maximised as FeAs_4_ adopts a nearly regular tetrahedron geometry, leading to an optimum *T*_c_^27^,^28^,^29^. The former is strongly related to the number and topology of Fermi surfaces, while the latter is due to the electron-phonon interaction. In agreement with the above rule, SmFeAsO_0.78_H_0.22_, which has the highest *T*_c_ of 55 K in iron pnictides, exhibits nearly ideal values of *α*_As-Fe-As_ (109.3°) and *h*_As_ (1.386 Å) at ambient pressure^30^. Moreover, the *α*_As-Fe-As_ for BaFe_2_As_2_ and LiFeAs act toward and away from the regular tetrahedron of FeAs_4_ along with increasing and decreasing the *T*_c_, respectively^21^,^22^. However, our present results reveal that while *α*_As-Fe-As_ and *h*_As_ of LaFeAsO_0.8_H_0.2_ deviate from the optimum values with pressure (Fig. 3), there is a significant increase in *T*_c_. Thus, this work highlights the inconsistencies in the guides for increasing the *T*_c_. The electronic state calculations illustrate that the Fermi surface topologies of LaFeAsO_0.8_H_0.2_ are unaltered on the application of pressure (Supplementary Information, Fig. S2). Additionally, the band width of Fe-3*d* widens with pressure because of the shortening of *d*_Fe-As_, resulting in a decrease of the spin-fluctuation that should cause *T*_c_ to decrease as well^27^. Thus, the properties examined so far fail to account for the increase of *T*_c_ under pressure.

**Figure 4:**
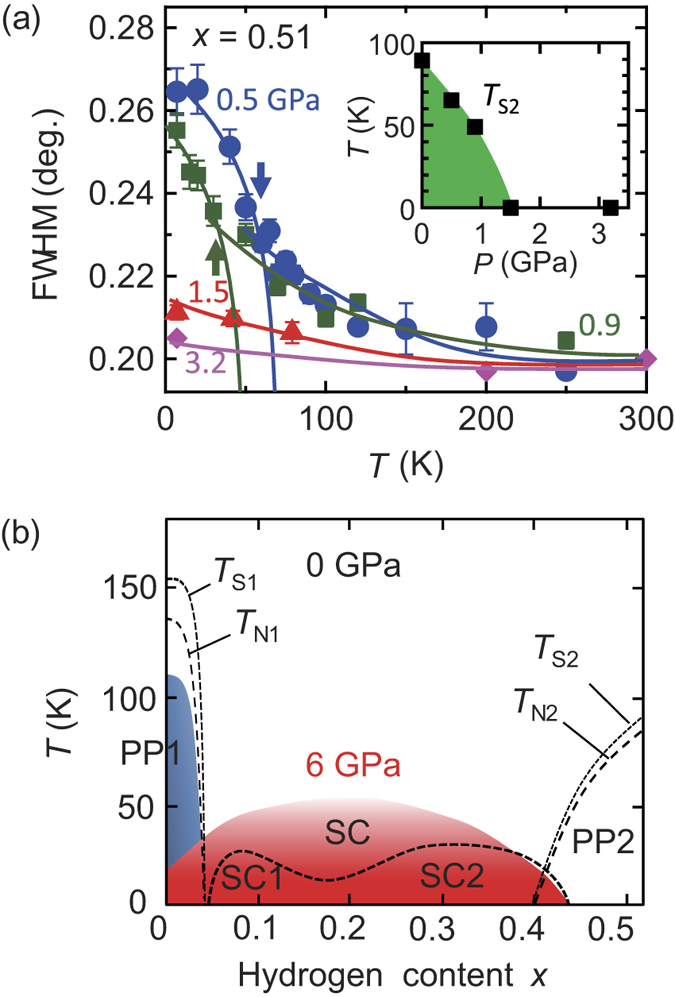
Full width at half maximum (FWHM) and electronic and structural phase diagram for LaFeAsO_1−*x*_H_*x*_. (**a**) The FWHM of the 220 reflection at several pressures for *x* = 0.51 estimated by the single quasi-Voigt function. The arrows indicate the T–O structural transitions of *T*_S2_. The solid lines are guides to the eyes. Inset shows *T*_S2_ as a function of pressure. (**b**) The *T* vs *x* phase diagram of the parent phases (PP) with structural (*T*_S_) and magnetic (*T*_N_) transitions, and superconducting phases (SC) at 0 and 6 GPa. The PP1 and SC at 6 GPa are blue and red, respectively. The error bars represent the uncertainty in the least-squares fitting of the peaks in (**a)**.

In order to find the reason behind the enhancement of *T*_c_, we next examined the parent phase under pressure. Both parent phases indicated tetragonal to orthorhombic (T–O) structural transitions (*T*_S_), and subsequently an antiferromagnetic ordering (*T*_N_). Figure 4a shows the temperature dependence of the full width at half maximum (FWHM) of the 220 reflection for *x* = 0.51, which is a good indicator of the T–O structural transition^11^. The transitions of *T*_S2_ are indicated by arrows and are estimated from the power law fitting. At 0.5 and 0.9 GPa, there is moderate broadening of the FWHM in the high-temperature region as extensive fluctuations occur in the vicinity of the structural transition. At 1.5 GPa, the FWHM exhibits slight broadening at low-temperature, not due to the structural transition but on account of the precursory phenomenon near the phase boundary. No broadening was observed at 3.2 GPa. Takahashi *et al*. demonstrated that an anomaly in resistivity, corresponding to *T*_N2_, for *x* = 0.51 was suppressed at ~2 GPa. This implies that the presence of *T*_N2_ as well as *T*_S2_, *i.e.* the PP2, vanished at low-pressure. Conversely, the PP1 could be seen up to 20 GPa (*T*_N1_) and 30 GPa (*T*_S1_) and was robust against pressure^15^,^16^,^17^. In Fig. 4b, we summarise the entire phase diagram of LaFeAsO_1−*x*_H_*x*_ at ambient pressure and 6 GPa.

The distinct responses to pressure for both parent phases are unexpected and presumably arise due to their different origins. Since the origin of PP1 is interpreted as a spin density wave with Fermi surface nesting^31^,^32^, its robust behaviour of PP1 against pressure is consistent with insensitive change of the Fermi surface^15^,^16^,^33^. In contrast, the origin of PP2 is seemingly less relevant to the Fermi surface topology. Iimura *et al*. performed an electronic state calculation based on the molecular orbital concept for LaFeAsO_1−*x*_H_*x*_^34^. In the low *x* region, the bonding and antibonding states of the Fe-3*d* and As-4*p* orbitals give a large gap. This gap decreases rapidly with an increase in *h*_As_, namely a decrease in the hybridization of Fe and As orbitals, making the non-bonding Fe-3*d*_xy_ orbital the half-filled state. The resulting state implies that the orbital selective Mott state on Fe-3*d*_xy_ is realised in the higher *x* region, *i.e.* in PP2, for LaFeAsO_1−*x*_H_*x*_^35^. Contrary to electron doping with increasing *x*, the pressure induces the rapid *decrease* of *h*_As_ as shown in Fig. 2c. Therefore, in contrast to PP1, PP2 can be easily broken by pressure.

Generally in high-*T*_c_ superconductors, the nature of the parent phase influences the superconducting state; that is, fluctuations derived from the parent phase may drive the pairing of the superconducting electrons. Thus, the origins of SC1 and SC2 adhering to PP1 and PP2, respectively, can be considered as fluctuations from PP1 and PP2^11^. The former is the itinerant type spin-fluctuation^32^, and the latter is another type spin-fluctuation with a strongly localised character of the orbital selective Mott phase. Since PP2 disappears easily with pressure, the fluctuation from PP2 should also be sensitive to pressure. Thus, we suggest that the significant enhancement of *T*_c_ under pressure is mainly due to the favourable change of the latter fluctuation. The relation between the present results and the other mechanism of orbital- or charge-fluctuations remains unclear^36^,^37^,^38^,^39^. To identify the above suggestion, the further investigation on the spin/structural dynamics is required in this system.

## Conclusion

We established that the FeAs_4_ tetrahedron in LaFeAsO_1−*x*_H_*x*_ deviates from the regular one with applied pressure, which is inconsistent with the conventional believed structural guides for increasing the *T*_c_. We found that the PP2 at *x* ~ 0.5 is lost under low-pressure, contrary to the sluggish pressure reaction of PP1 at *x* ~ 0. Pressure presumably suppresses the itinerant spin-fluctuation driving the superconductivity from PP1, but enhances another spin-fluctuation with the localised character from PP2 in 1111-type iron pnictides. These pressure responses of the FeAs_4_ modification and the parent phases are previously unexplained peculiarities in 1111-type. In future, we plan to further increase the *T*_c_ by combining the effects of the regular tetrahedron geometry of FeAs_4_ and the strong fluctuation state from PP2 under pressure in 1111 systems.

## Methods

LaFeAsO_1−*x*_H_x_ powder samples were prepared by a high-pressure solid-state reaction as reported in literature^7^. The hydrogen content in these systems was determined by thermal desorption spectroscopy. Synchrotron X-ray diffraction (sXRD) was performed at room temperature for pressures up to ~8 GPa at NE1A of PF-AR, KEK. Pressure was applied using a diamond anvil cell (DAC) with 600 μm culet diamond anvils and a gasket with a 300 μm hole. The DAC, in which the anvils are supported by the 45° tapered slit window without a backing plate, has the advantage of reducing the background scatter and simplifying the absorption correction of diamond (Supplementary Information, Fig. S3a). A very fine powder results in a perfect Debye ring of reflections on the two-dimensional image (Supplementary Information, Fig. S3b). The measured wavelength of λ = 0.4217 Å and the wide open angle of the DAC can approach a *d*-spacing of <0.7 Å, which enables accurate determination of atomic positions. The two-dimensional images collected using a RIGAKU R-AXIS on the curved imaging plate were integrated to yield 2*θ*-intensity data. Crystal structures were refined by the Rietveld method indexed to the space group of *P*4/*nmm*, and the reliable factors in the refinements were *R*_wp_ = 3.9–6.6% (Supplementary Information, Fig. S3c)^40^. Moreover, we traced the PP2 on the application of pressure by means of sXRD down to 8 K at BL-8B of PF, KEK, measured at λ = 0.8267 Å. A 4:1 methanol-ethanol mixture was used as a pressure transmitting medium in all the experiments.

## Additional Information

**How to cite this article**: Kobayashi, K. *et al*. Pressure effect on iron-based superconductor LaFeAsO_1−*x*_H*_x_*: Peculiar response of 1111-type structure. *Sci. Rep.* **6**, 39646; doi: 10.1038/srep39646 (2016).

**Publisher's note:** Springer Nature remains neutral with regard to jurisdictional claims in published maps and institutional affiliations.

## Supplementary Material

Supplementary Information
