# Supplementary material for: Pressure effect on iron-based superconductor LaFeAsO1−xHx: Peculiar response of 1111-type structure
Source: Sci Rep. 2016 Dec 22;6:39646. doi: 10.1038/srep39646 (PMC5177881; doi:10.1038/srep39646)
Supplement: Supplementary Information [file srep39646-s1.pdf]

Supplementary Information for

Pressure effect on iron-based superconductor $LaFeAsO_{1-x}H_x$: Peculiar response of 1111-type structure

Kensuke Kobayashi[1], Jun-ichi Yamaura[2], Soshi Iimura[3], Sachiko Maki[2], Hajime Sagayama[1], Reiji Kumai[1, 4], Youichi Murakami[1, 4], Hiroki Takahashi[5], Satoru Matsuishi[2], and Hideo Hosono[2, 3]

[1]Institute of Materials Structure Science, High Energy Accelerator Research Organization (KEK), Tsukuba, Ibaraki 305-0801, Japan

[2]Materials Research Center for Element Strategy, Tokyo Institute of Technology, Yokohama, Kanagawa 226-8503, Japan

[3]Laboratory for Materials and Structures, Tokyo Institute of Technology, Yokohama, Kanagawa 226-8503, Japan

[4]Department of Materials Structure Science, The Graduate University for Advanced Studies, Tsukuba, Ibaraki 305-0801, Japan

[5]College of Humanities and Science, Nihon University, Setagaya, Tokyo 156-8550, Japan

Lattice constants and structural parameters ($d_{Fe\text{-}As}$, $\alpha_{As\text{-}Fe\text{-}As}$, h_{As}, $d_{La\text{-}As}$) for $LaFeAsO_{1\text{-}x}H_x$ (x = 0, 0.20, 0.51).

Table S1 Detailed experimental data of the lattice constants, the structural parameters ($d_{Fe\text{-}As}$, $\alpha_{As\text{-}Fe\text{-}As}$, h_{As}, $d_{La\text{-}As}$) for $LaFeAsO_{1\text{-}x}H_x$ (x = 0, 0.20, 0.51) under pressure.

x =0

P (GPa)	a (Å)	c (Å)	$d_{Fe\text{-}As}$(Å)	$\alpha_{As\text{-}Fe\text{-}As}$ (°)	h_{As} (Å)	$d_{La\text{-}As}$ (Å)
0	4.0351(1)	8.7393(5)	2.411(1)	113.63(9)	1.319(2)	3.383(1)
0.5	4.0286(2)	8.7121(6)	2.405(2)	113.73(10)	1.315(4)	3.371(1)
1.1	4.0211(1)	8.6779(4)	2.402(2)	113.65(9)	1.314(3)	3.359(1)
2.5	4.0068(2)	8.6151(7)	2.389(3)	113.98(12)	1.302(5)	3.341(1)
3.3	3.9991(1)	8.5743(5)	2.386(3)	113.88(11)	1.301(4)	3.325(1)
4.4	3.9864(2)	8.5165(6)	2.377(2)	113.97(10)	1.295(4)	3.306(1)
5.5	3.9775(2)	8.4725(6)	2.374(2)	113.83(10)	1.297(3)	3.293(1)

x = 0.20

P (GPa)	a (Å)	c (Å)	$d_{Fe\text{-}As}$(Å)	$\alpha_{As\text{-}Fe\text{-}As}$ (°)	h_{As} (Å)	$d_{La\text{-}As}$ (Å)
0	4.0113(1)	8.7026(4)	2.421(2)	111.86(10)	1.356(3)	3.308(1)
0.5	4.0024(1)	8.6653(4)	2.414(2)	112.01(9)	1.350(3)	3.299(1)
1.1	3.9986(1)	8.6493(4)	2.411(1)	112.07(9)	1.347(2)	3.294(1)
1.9	3.9908(1)	8.6102(4)	2.404(1)	112.24(8)	1.340(2)	3.284(1)
2.5	3.9845(1)	8.5799(4)	2.397(1)	112.47(9)	1.332(2)	3.276(1)
3.0	3.9794(1)	8.5523(4)	2.393(2)	112.52(9)	1.329(3)	3.268(1)
3.6	3.9751(1)	8.5312(4)	2.388(2)	112.70(9)	1.323(3)	3.263(1)
4.7	3.9658(1)	8.4849(4)	2.380(2)	112.82(10)	1.318(3)	3.248(1)
5.2	3.9641(1)	8.4720(4)	2.378(2)	112.95(9)	1.313(3)	3.247(1)
7.7	3.9416(1)	8.3558(4)	2.361(1)	113.15(9)	1.301(3)	3.211(1)

x = 0.51

P (GPa)	a (Å)	c (Å)	$d_{Fe\text{-}As}$(Å)	$\alpha_{As\text{-}Fe\text{-}As}$ (°)	h_{As} (Å)	$d_{La\text{-}As}$ (Å)
0	3.9757(1)	8.6528(1)	2.439(1)	109.19(7)	1.413(2)	3.205(1)
0.7	3.9652(1)	8.6073(4)	2.424(2)	109.73(9)	1.395(3)	3.195(1)
2.2	3.9473(1)	8.5235(4)	2.405(2)	110.27(10)	1.375(3)	3.178(1)
3.6	3.9343(1)	8.4616(3)	2.340(2)	110.11(9)	1.374(3)	3.160(1)
4.4	3.9295(1)	8.4363(3)	2.393(2)	110.39(9)	1.366(3)	3.155(1)
5.1	3.9229(1)	8.4030(4)	2.382(2)	110.85(9)	1.352(3)	3.153(1)
6.0	3.9188(1)	8.3803(3)	2.382(2)	110.71(19)	1.354(3)	3.145(1)

X-ray profiles and atomic positions

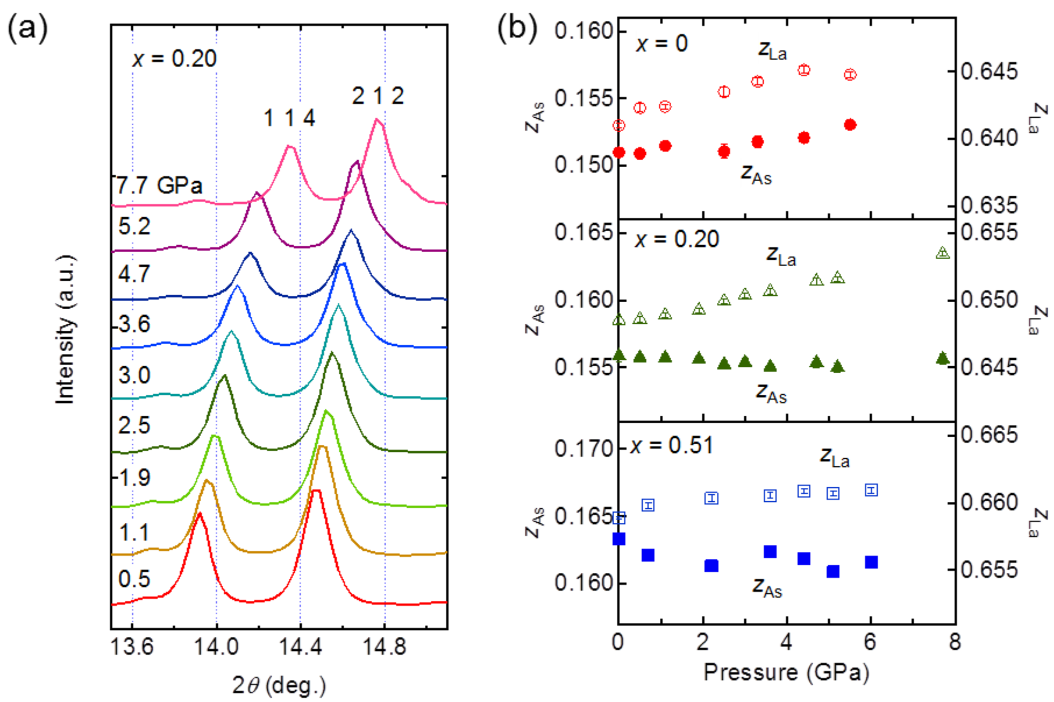

Figure S1 (**a**) X-ray profiles at several pressures for x = 0.20. (**b**) Atomic positions of z_{As} for As (left axis) and z_{La} for La (right axis). Each atoms are located on the 2c site (1/4, 1/4, z_{La}) for La, 2a (3/4, 1/4, 0) for Fe, 2c (1/4, 1/4, z_{As}) for As, and 2b (3/4, 1/4, 1/2) for O in the tetragonal system and the space group of *P*4/*nmm* (No.129, origin choice 2). The error bars represent the uncertainly in the least-squares fitting of the whole patterns in **a**.

Band structure calculation

The electronic states were calculated based on the obtained crystal parameters using WIEN2K code, and the effect of electron-doping was approximated by substituting the oxygen (Z = 8) into virtual atoms that have a fractional nuclear charge (Z = 8 + 0.2). Figure S2a shows electronic structures of $LaFeAsO_{0.8}H_{0.2}$ at 0 and 7.7 GPa. The 10 bands near the Fermi level are mainly derived from Fe 3d electrons. Pressure gives rise to the bands elevated and depressed away from the Fermi level, and the width of each band broadened owing to increasing the Fe-3d and As-4p hybridization with the $d_{Fe\text{-}As}$ shortening and the h_{As} lowering. Moreover, there is no pronounced difference of the Fermi surface topologies at 0 and 7.7 GPa as depicted in Figs. S2b and 2c except for the tiny expansion of the area near the Z point.

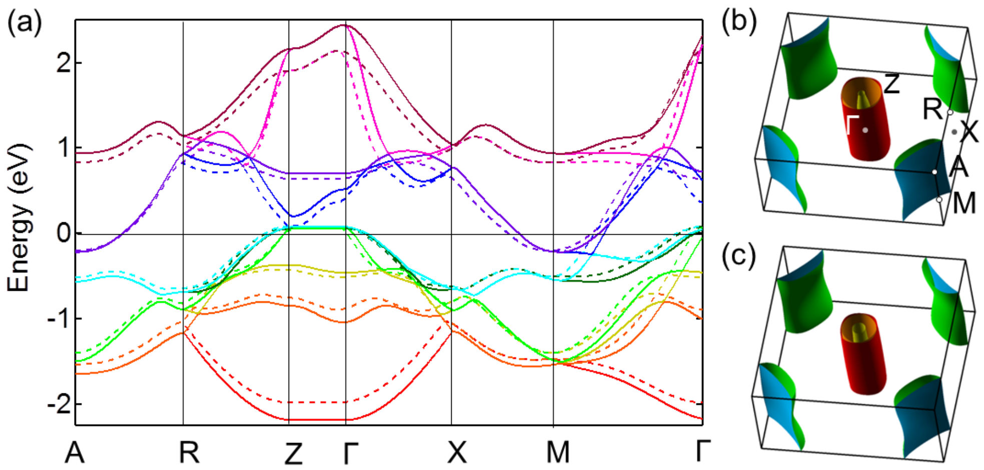

Figure S2 Calculated electronic structures of $LaFeAsO_{0.8}H_{0.2}$. (**a**) Energy-band structures are depicted by the dotted and solid lines for 0 and 7.7 GPa, respectively. Fermi surfaces at 0 GPa in (**b**) and 7.7 GPa in (**c**).

Structure determination

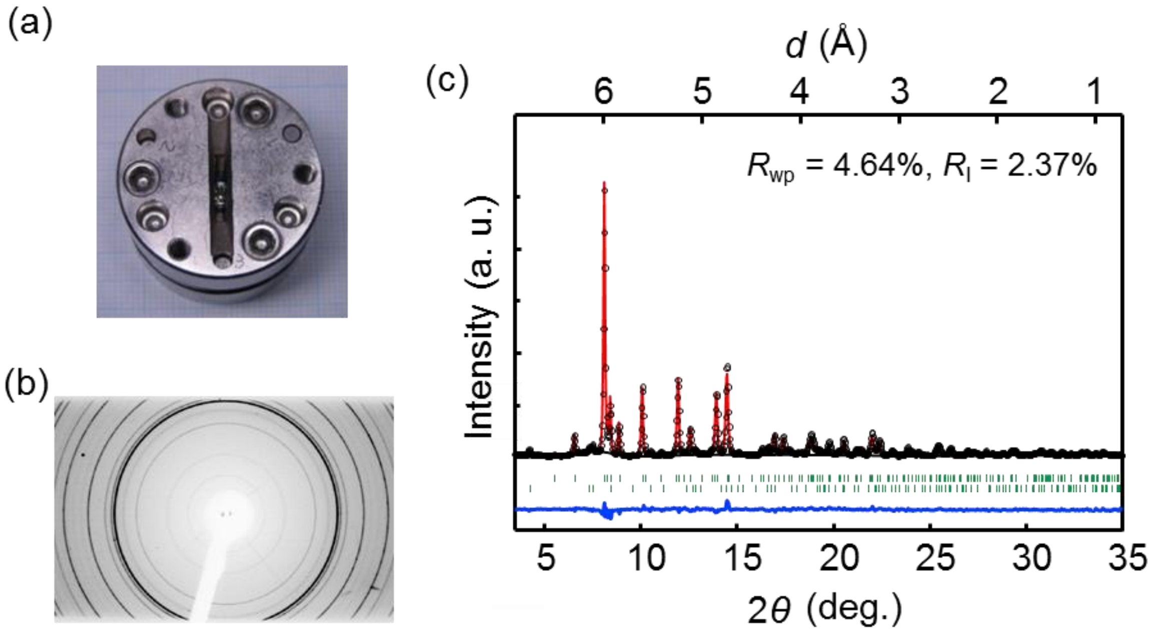

Figure S3 (**a**) Photograph of the slit type DAC. The diamond anvils are supported by the 45° tapered slit window without the backing plate, enables the data collection to $2\theta < 45°$. (**b**) Debye-Scherrer ring on two-dimensional image. (**c**) Refined powder X-ray diffraction pattern of $LaFeAsO_{0.8}H_{0.2}$ at 7.7 GPa. The observed (black circle), calculated (red line), difference profiles (blue line), and positions of Bragg peaks (green ticks) are plotted. Lower ticks represent the impurity of $La(OH)_3$.
